# A genome wide survey reveals multiple nematocyst-specific genes in Myxozoa

**DOI:** 10.1186/s12862-018-1253-7

**Published:** 2018-09-12

**Authors:** Erez Shpirer, Arik Diamant, Paulyn Cartwright, Dorothée Huchon

**Affiliations:** 1 School of Zoology, Tel Aviv University, Tel Aviv, Israel; 2 National Center for Mariculture, Israel Oceanographic and Limnological Research, Eilat, Israel; 3 Department of Ecology and Evolutionary Biology, University of Kansas, Lawrence, USA; 4 The Steinhardt Museum of Natural History and National Research Center, Tel Aviv University, Tel Aviv, Israel

**Keywords:** Cnidaria, Taxonomically restricted genes, Polar capsule, Phylogeny, Endoparasitism

## Abstract

**Background:**

Myxozoa represents a diverse group of microscopic endoparasites whose life cycle involves two hosts: a vertebrate (usually a fish) and an invertebrate (usually an annelid worm). Despite lacking nearly all distinguishing animal characteristics, given that each life cycle stage consists of no more than a few cells, molecular phylogenetic studies have revealed that myxozoans belong to the phylum Cnidaria, which includes corals, sea anemones, and jellyfish. Myxozoa, however, do possess a polar capsule; an organelle that is homologous to the stinging structure unique to Cnidaria: the nematocyst. Previous studies have identified in Myxozoa a number of protein-coding genes that are specific to nematocytes (the cells producing nematocysts) and thus restricted to Cnidaria. Determining which other genes are also homologous with the myxozoan polar capsule genes could provide insight into both the conservation and changes that occurred during nematocyst evolution in the transition to endoparasitism.

**Results:**

Previous studies have examined the phylogeny of two cnidarian-restricted gene families: minicollagens and nematogalectins. Here we identify and characterize seven additional cnidarian-restricted genes in myxozoan genomes using a phylogenetic approach. Four of the seven had never previously been identified as cnidarian-specific and none have been studied in a phylogenetic context. A majority of the proteins appear to be involved in the structure of the nematocyst capsule and tubule. No venom proteins were identified among the cnidarian-restricted genes shared by myxozoans.

**Conclusions:**

Given the highly divergent forms that comprise Cnidaria, obtaining insight into the processes underlying their ancient diversification remains challenging. In their evolutionary transition to microscopic endoparasites, myxozoans lost nearly all traces of their cnidarian ancestry, with the one prominent exception being their nematocysts (or polar capsules). Thus nematocysts, and the genes that code for their structure, serve as rich sources of information to support the cnidarian origin of Myxozoa.

**Electronic supplementary material:**

The online version of this article (10.1186/s12862-018-1253-7) contains supplementary material, which is available to authorized users.

## Background

Myxozoa are microscopic parasites that principally infect fish, annelids, and Bryozoa [1]. Their spores are characterized by the presence of complex organelles, called polar capsules, which are triggered during host infection and are thought to assist in attachment to the host tissue. Myxozoa were originally described as protists [2], but for over 100 years there has been the suggestion that they show an affinity to Cnidaria, which includes jellyfish and corals, [3]. This suggestion is based on the observation that myxozoan polar capsules bear a remarkable similarity to the cnidarian stinging structures (i.e., the nematocysts) [4]. Molecular phylogenetic studies have confirmed that myxozoans are cnidarians [5] and a probable sister clade to Medusozoa (hydras, jellyfishes) [6–9]. Myxozoa, which are composed of only one or a few cells, have lost most of their cnidarian characteristics, except for the nematocyst (called the polar capsule in Myxozoa) [9]. This complex structure is thus the only prominent feature that unites all members of the phylum Cnidaria [5, 10].

In a previous paper, Shpirer et al. [11] demonstrated that the nematocyst-restricted structural protein families, minicollagen and nematogalectin, are present in Myxozoa. This finding strengthened the hypothesis that myxozoans are cnidarians and that the polar capsule is the nematocyst homolog in Myxozoa [11]. Thus, comparative investigations of the molecular components underlying nematocysts, and particularly the genes restricted to Cnidaria, could contribute to better understanding the evolution of this diverse phylum [12], including its transition from a free-living cnidarian to a microscopic endoparasite.

Previous investigations have identified a number of cnidarian-restricted, nematocyst-specific genes and proteins [13–17]. In this study, we aimed to determine whether myxozoans possess nematocyst-specific proteins (other than nematogalectins and minicollagens, which have been characterized in Myxozoa in several works [6, 11, 15, 17–19]) to better understand the origins and evolution of myxozoan polar capsules from cnidarian nematocysts. Using a database of nematocyst-specific proteins generated through a *Hydra* nematocyst proteome sequencing [14], we searched myxozoan and other cnidarian genomic and transcriptomic databases for proteins with similar sequences. We then took a phylogenetic approach to identify and further characterize myxozoan homologs of cnidarian-restricted nematocyst proteins.

## Results

### Identification of nematocyst-specific cnidarian-restricted proteins shared between cnidarians and Myxozoa

Reciprocal BLAST (Basic Local Alignment Search Tool) searches [20] were initially performed using *Hydra vulgaris* (syn. *Hydra magnipapillata*) nematocyst proteins [14] as queries to identify nematocyst-restricted genes in the genome and transcriptome of the myxozoan *Kudoa iwatai* (see Methods). Phylogenetic reconstructions were then executed for each candidate using cnidarian and non-cnidarian homologs identified by BLAST searches. We considered as nematocyst-specific genes, those for which the *Hydra* genes identified from the nematocyst proteome [14] and the *Kudoa* candidate homologs belonged to monophyletic clades that encompass only cnidarian representatives. We identified seven such proteins, not including nematogalectins and minicollagens, which had been characterized in other studies [11, 18]. The seven proteins are hereby termed nematocyst-specific proteins, or NSP1–7.

Balasubramanian et al. [14] classified nematocyst proteins identified in *Hydra* according to their domain annotations. Three out of the seven genes we characterized had been annotated as structural proteins by Balasubramanian et al. [14], one was a serine peptidase, one was a metabolic glutamate enzyme and the last two they did not annotate and were designated as novel. Balasubramanian et al. [14] also categorized shell proteins, defined as those proteins that are insoluble proteins associated with the nematocyst capsule wall and tubule structure. Four out of the seven genes characterized here were classified as shell proteins [14] (Table 1). The structure and phylogenetic relationships of each protein are described below.

**Table 1:** Summary of NSP gene features

	NSP1	NSP2	NSP3	NSP4	NSP5	NSP6	NSP7
Orphan gene^a^	X	X	X	X	X	–	–
Conserved domains	TSP1, LamG3	LamG3	Galectin	CRD	MANEC	Peptidase S8	γ glutamyl transpeptidase
Single copy	–	X	X	–	X	–	–
Previously reported	–	X (nb012)	–	–	–	X	X
Signal peptide in NSP	X	X	X	X	–	X	–
Transmembrane domain	–	–	–	–	X	–	X
Protein category^b^	Structural	Structural	Structural	Novel	Novel	Serine peptidase	Metabolic enzyme glutamate
Shell Protein^b^	X	X	–	X	–	–	X
Higher copy number in Hydrozoa compared to anthozoans	–	–	X	–	–	X	–
Present in *C. shasta* polar capsule proteome	X	X	X	–	–	X	X
Cnidarian clades found to possess the gene	Anthozoa Medusozoa Myxozoa	Anthozoa Medusozoa Myxozoa *Polypodium*	Medusozoa Myxozoa *Polypodium*	Anthozoa Medusozoa Myxozoa *Polypodium*	Anthozoa Medusozoa Myxozoa *Polypodium*	Anthozoa Medusozoa Myxozoa *Polypodium*	Anthozoa Medusozoa Myxozoa *Polypodium*

^a^ no known homolog outside of Cnidaria

^b^ as annotated by Balasubramanian et al. [14]

Several branches in our phylogenetic reconstructions (described below) received low support. This low support can be explained by saturation (Cnidaria is an ancient lineage, which diversified in the Precambrian or Early Cambrian [21, 22]) and by the fact that all these reconstructions are based on a single gene. Nevertheless, our goal here was not to reconstruct cnidarian relationships but rather, to detect nematocyst-specific proteins. This can be inferred despite the low bootstrap support within cnidarian clades, since the critical node in each phylogenetic tree is the one supporting the monophyly of the nematocyst-specific proteins. In each case, this node was well supported by (BP > 95 and PP = 1).

### NSP1

#### Structure

**Fig. 1:**
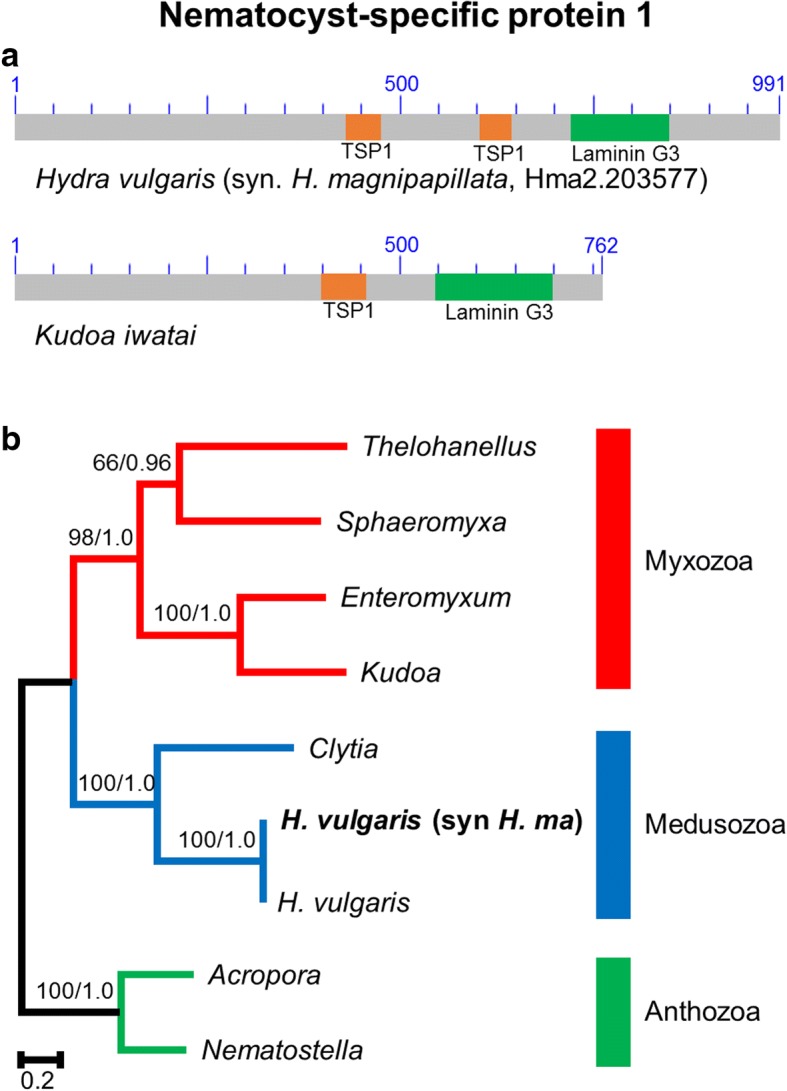
Nematocyst-specific protein 1 domain architecture and ML phylogenetic tree. **a**. Schematic drawing of the domain architecture of NSP1 for *Hydra magnipapillata* and *Kudoa iwatai*. For each domain the CDD definition is given. **b**. ML phylogenetic tree reconstructed using the NSP1 dataset. ML bootstrap (BP_ML_)/Bayesian posterior probabilities supports are given for nodes with BP_ML_ above 50%. Red, blue and green represent Myxozoa, Medusozoa, and Anthozoa respectively. The original *H. vulgaris* (syn. *H. magnipapillata*) protein appears in bold. Because non-cnidarian homologs do not exist the tree was rooted with anthozoan sequences

The NSP1 gene possesses one or two thrombospondin type-1 domains (TSP1) followed by a laminin G3-like domain (LamG) (Fig. 1a). The TSP1 domain is present in adhesive glycoproteins usually found to mediate cell-to-cell and cell-to-matrix interactions [23]. The laminin G3-like domain is usually found in the extracellular proteins that form a major component of the basal lamina [24, 25]. No gene with this domain structure has been found outside of cnidarians. The LamG domain was found in all of the cnidarian sequences recovered in the BLAST searches. The number of TSP1 domains, however, varied among those sequences. This is probably due to sequence incompleteness or difficulties in identifying domains due to higher rates of sequence evolution at the 3′ end. Two TSP1 domains were found in *H. magnipapillata*, *H. vulgaris* and *Sphaeromyxa zaharoni*. One TSP1 domain was found in *K. iwatai* and *Nematostella vectensis* and no TSP1 domains were recovered in *Acropora millepora*, *Clytia hemisphaerica*, *Enteromyxum leei*, and *Thelohanellus kitauei*. A signal peptide was found only for the two anthozoans, *A. millepora* and *N. vectensis*.

#### Phylogeny

Homologs of NSP1 were found in representatives of each of the three major clades of Cnidaria (i.e., Anthozoa, Medusozoa and Myxozoa) (Fig. 1b). The phylogenetic tree of the NSP1 gene agrees with the commonly accepted view of cnidarian relationships [9, 11, 26] and each clade has high support (BP_ML_ = 98–100, PP = 1.0). The relationships within myxozoans followed the commonly accepted view [27] with a deep division between a marine clade (*Kudoa* and *Enteromyxum*) and a freshwater clade (*Thelohanellus* and *Sphaeromyxa*).

### NSP2

#### Structure

**Fig. 2:**
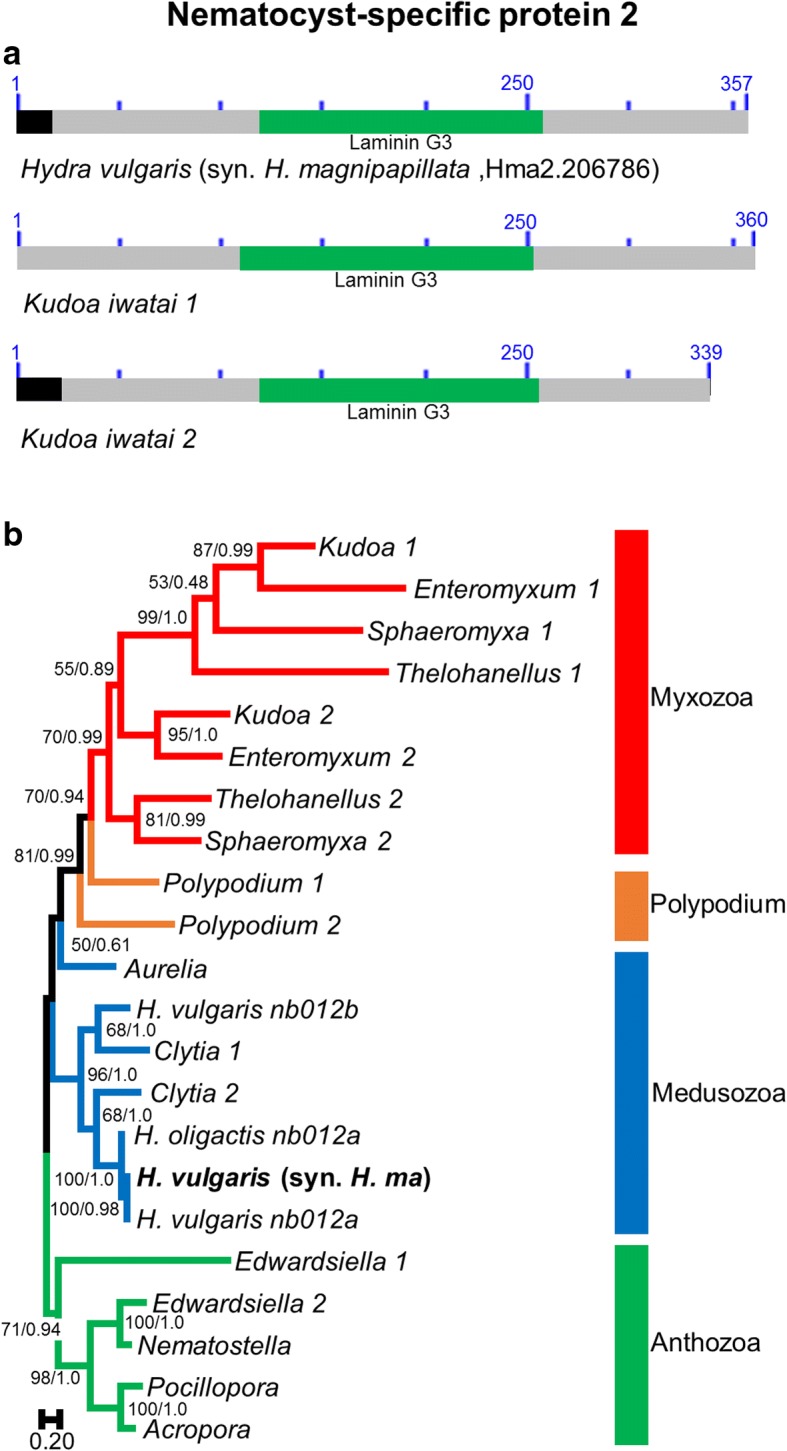
Nematocyst-specific protein 2 domain architecture and ML phylogenetic tree. **a**. Schematic drawing of the domain architecture of NSP2 for *Hydra magnipapillata* and *Kudoa iwatai*. For each domain the CDD definition is given. Signal peptides found are colored in black. **b**. ML phylogenetic tree reconstructed using the NSP2 dataset. ML bootstrap (BP_ML_)/Bayesian posterior probabilities supports are given for nodes with BP_ML_ above 50%. Red, orange, blue and green represent Myxozoa, *Polypodium*, Medusozoa, and Anthozoa respectively. The original *H. vulgaris* (syn. *H. magnipapillata*) protein appears in bold. Because non-cnidarian homologs do not exist the tree was rooted with anthozoan sequences

NSP2 proteins have previously been identified in *H. vulgaris* and been termed nematoblast-specific protein 12 (nb012) [16] (Fig. 2a). Specifically, two transcripts, termed *nb012a* and *nb012b*, were identified. They are identical in their 5′-regions but differ in their 3′-end [16]. The *Hydra* genome assembly shows that these two transcripts originated through an alternative splicing of the same gene. Specifically, one of the 5′-exons is shared between the two transcripts while all other exons belong to different DNA regions (NW_004173076 [28]). Although the 3’ends differ, they reveal similar amino-acid sequences. It is thus likely that these two transcripts originated through a tandem duplication of the 3′-exons.

This conserved protein possesses a single Laminin G3-like domain and does not demonstrate a close homology (i.e. e-value below 1e-05) to other, non-cnidarian, animal proteins. While for *Aurelia aurita*, *Hydra oligactis*, *A. millepora*, *Pocillopora damicornis*, and *N. vectensis* we only found one transcript, other cnidarians were found to possess two transcript copies. Because the 5′-ends (the region that is shared between the two *Hydra* transcripts) are often incomplete we could not determine whether these transcripts belong to the same or to duplicated genes. All sequences possessed the Laminin G3-like domain, except the one from *Thelohanellus kitauei*, which was truncated. A signal peptide was found for all sequences, except those that have a truncated 5′ end.

#### Phylogeny

Homologs of NSP2 were found in representatives of each of the three major clades of Cnidaria (i.e., Anthozoa, Medusozoa, and Myxozoa), as well as the parasitic sister taxon to myxozoans, *Polypodium* (Fig. 2b). Although two transcripts were found for most species, the NSP2 phylogenetic tree is not divided into two different clades. Rather, the tree is divided into three major clades: Anthozoa (BP = 71; PP = 0.94), Hydrozoa (BP = 96; PP = 1.0), and Endocnidozoa (Polypodium + Myxozoa) (BP = 81; PP = 0.99), with each containing both NSP2 genes. The position of the *Aurelia* protein is clustered with low support value (BP = 50; PP = 0.61) with Endocnidozoa. This gene tree topology suggests that the NSP2 protein family has been evolving under some level of concerted evolution, as assuming numerous independent duplications is less likely. Concerted evolution is favored when genes are tandemly duplicated, which agrees with the structure of the gene observed in *Hydra*. Sequence homogenization is however not total, since within a species the two NSP2 genes are usually not closely related. For example, *Enteromyxum* 1 is closely related to *Kudoa* 1 rather than to *Enteromyxum* 2 (Fig. 2b).

### NSP3

#### Structure

**Fig. 3:**
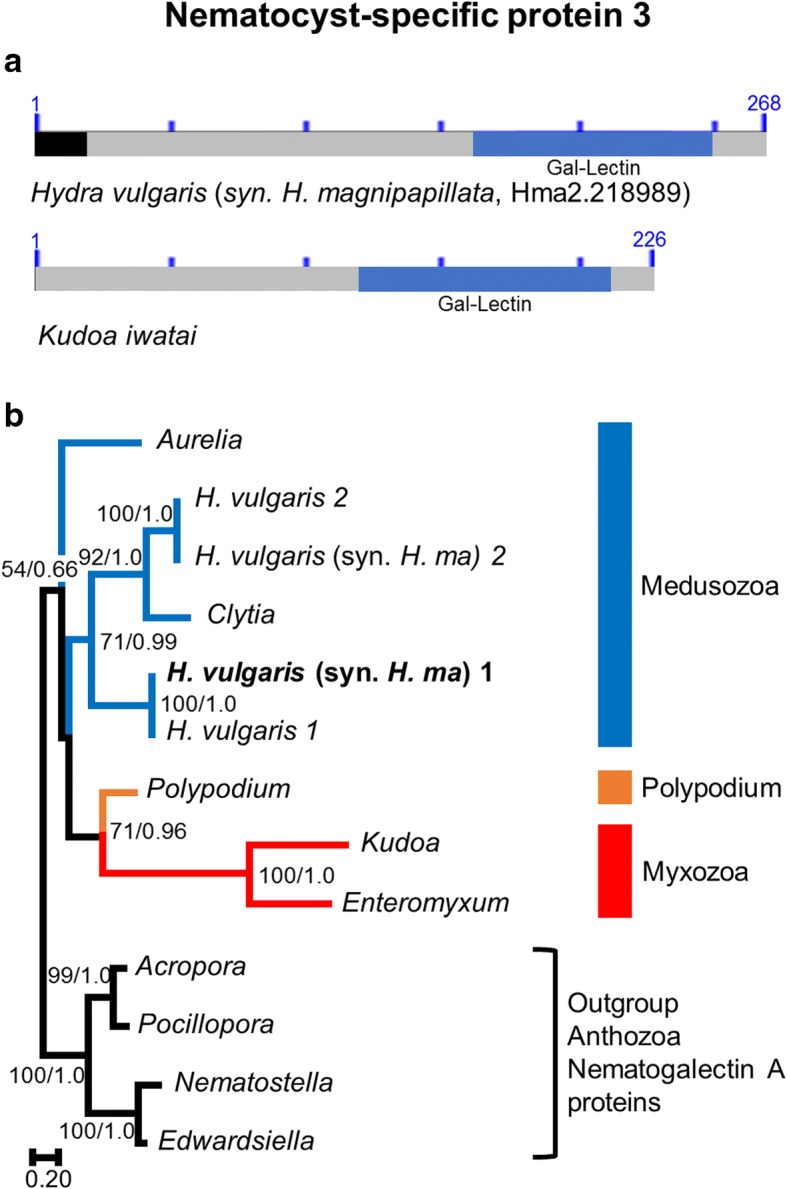
Nematocyst-specific protein 3 domain architecture and ML phylogenetic tree. **a**. Schematic drawing of the domain architecture of NSP3 for *Hydra magnipapillata* and *Kudoa iwatai*. For each domain the CDD definition is given. Signal peptides found are colored in black. **b**. ML phylogenetic tree reconstructed using the NSP3 dataset. ML bootstrap (BP_ML_)/Bayesian posterior probabilities supports are given for nodes with BP_ML_ above 50%. Red, orange, and blue represent Myxozoa, *Polypodium* and Medusozoa respectively. The original *H. vulgaris* (syn. *H. magnipapillata*) protein appears in bold. Because non-cnidarian homologs do not exist the tree was rooted with anthozoan sequences

NSP3 proteins all have a single galactose-binding lectin domain (galectin domain) (Fig. 3a). The galectin family proteins are involved in cell–cell interactions, cell–matrix adhesion and transmembrane signaling [29]. BLAST searches show that the galectin domain of NSP3 is closely related to the galectin domain of anthozoan nematogalectin A, and only distantly related to non-cnidarian galectin domains. However the NSP3 protein lacks the collagen domain that characterizes nematogalectins [11, 15]. Since nematogalectins are involved in the nematocyst structure it is probable that NSP3 has a similar function. Interestingly, NSP3 was found to be duplicated in *Hydra*.

#### Phylogeny

Homologs of NSP3 proteins have been found in myxozoans, *Polypodium,* and medusozoans but not in anthozoans (Fig. 3b). Basal relationships among NSP3 sequences are not well resolved, and the monophyly of medusozoans is not recovered. The monophyly of Hydrozoa and Endocnidozoa is only moderately supported (BP = 71 PP = 0.99, 0.96 respectively). Interestingly, the *Hydra* NSP3–2 is more closely related to the *Clytia* protein than to *Hydra* NSP3–1, suggesting a duplication in the branch leading to *Clytia* and *Hydra*.

### NSP4

#### Structure

**Fig. 4:**
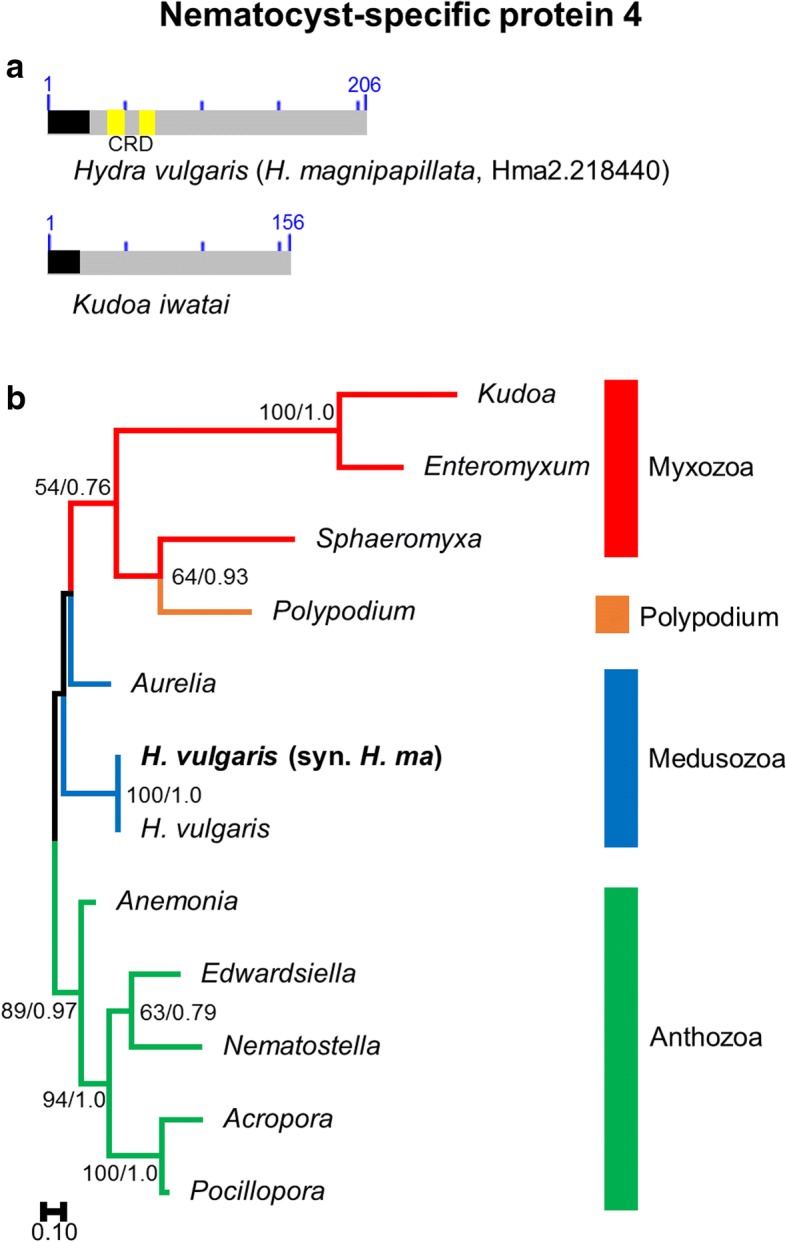
Nematocyst-specific protein 4 domain architecture and ML phylogenetic tree. **a**. Schematic drawing of the domain architecture of NSP4 for *Hydra magnipapillata* and *Kudoa iwatai*. No domain homology was found in the CDD. Yellow represents the cysteine-rich domain (CRD) indicated by Balasubramanian et al. [3]. Signal peptides found are colored in black. **b**. ML phylogenetic tree reconstructed using the NSP4 dataset. ML bootstrap (BP_ML_)/Bayesian posterior probabilities supports are given for nodes with BP_ML_ above 50%. Red, orange, blue and green represent Myxozoa, *Polypodium*, Medusozoa, and Anthozoa respectively. The original *H. vulgaris* (syn. *H. magnipapillata*) protein appears in bold. Because non-cnidarian homologs do not exist the tree was rooted with anthozoan sequences

NSP4 proteins are characterized by highly conserved 3′-ends (Fig. 4a). This region, however, was not found to correspond to any known protein domain following a search on the NCBI conserved domain (CDD) search webserver. Similarly, the 3′ end region is not shared by any known protein (i.e., when conducting blastp searches E-values >1e-5). The 5′-end of the sequence is both cysteine and proline rich in all cnidarian lineages except Myxozoa and *Polypodium*. Proline-rich domains are often involved in protein-protein interaction [30] and are known to have an important impact on protein structure. Balasubramanian et al. [14] indicated the presence of a cysteine-rich-domain (or CRD) in the sequence of *Hydra*. Interestingly, CRDs also characterize minicollagens and other important structural nematocyst proteins [11, 31–33]. Specifically, the CRDs of minicollagens are known to polymerize to form the basic scaffold of the nematocyst capsule [32, 34, 35]. A signal peptide was identified for all sequences that were not truncated at their 5’end.

#### Phylogeny

Homologs of NSP4 were found in representatives of all major clades of Cnidaria (i.e., Anthozoa, Medusozoa, Myxozoa and *Polypodium*) (Fig. 4b). The phylogenetic relationships among NSP4 proteins globally agrees with the commonly accepted view of cnidarian relationships [5, 30], with three exceptions. First, *Aurelia* is recovered as the sister clade of Endocnidozoa rather than of *Hydra*, but with very low support (BP < 50; PP < 0.5). *Polypodium* is placed as the sister clade of *Sphaeromyxa* rather than of Myxozoa but also with low support (BP < 70; PP < 0.95)*.* Finally, *Anemonia* is the first diverging anthozoan lineage rather than the sister clade of *Nematostella* and *Edwardsiella*, with rather high support (BP > 80; PP > 0.95). It should be noted that *Anemonia* and *Sphaeromyxa* have truncated sequences that could obscure their phylogenetic placement.

### NSP5

#### Structure

**Fig. 5:**
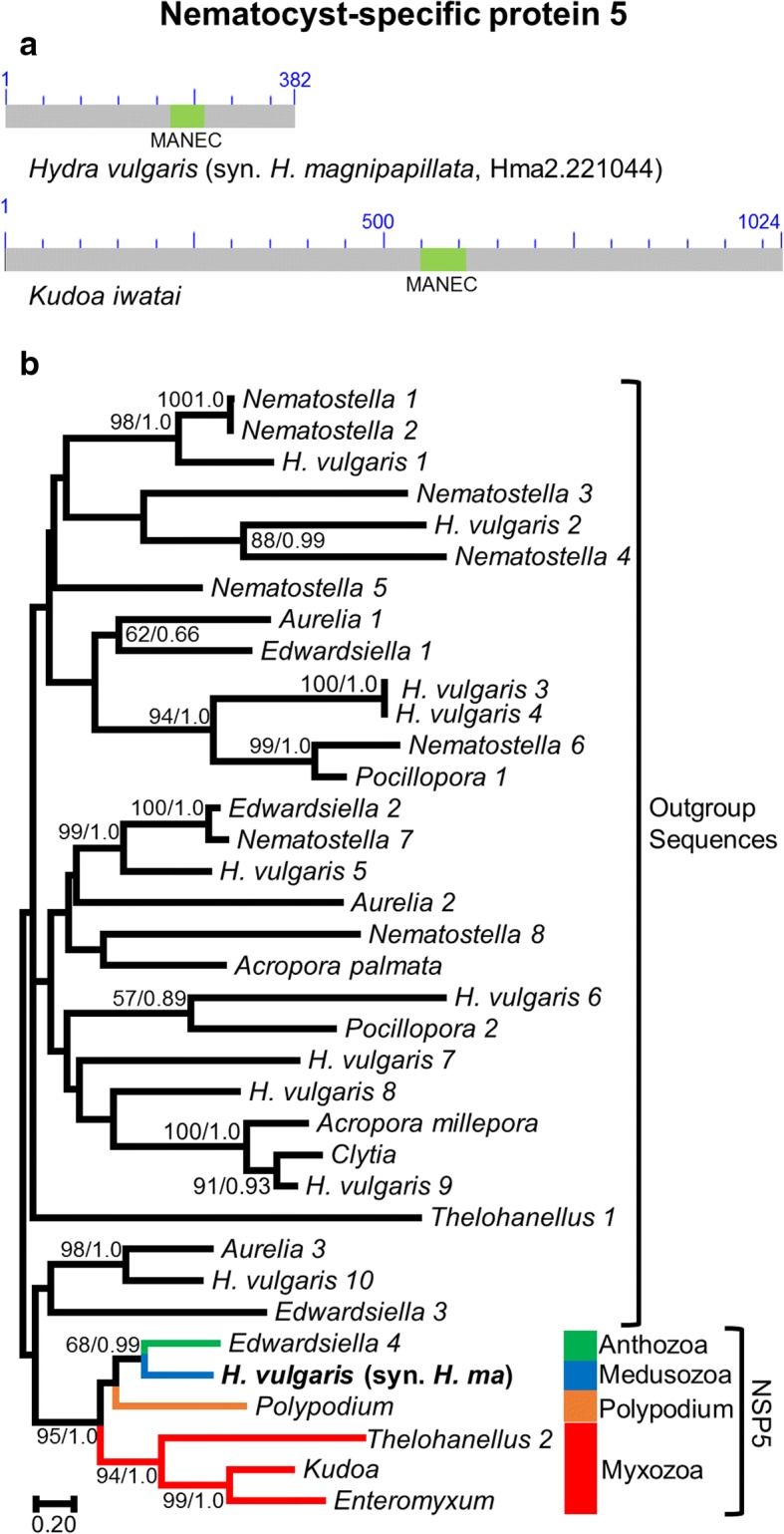
Nematocyst-specific protein 5 domain architecture and ML phylogenetic tree. **a**. Schematic drawing of the domain architecture of NSP5 for *Hydra magnipapillata* and *Kudoa iwatai*. For each domain the CDD definition is given. **b**. ML phylogenetic tree reconstructed using the NSP5 dataset. ML bootstrap (BP_ML_)/Bayesian posterior probabilities supports are given for nodes with BP_ML_ above 50%. Red, orange, blue and green represent Myxozoa, *Polypodium*, Medusozoa, and Anthozoa respectively. The original *H. vulgaris* (syn. *H. magnipapillata*) protein appears in bold. The tree was rooted with distant cnidarian proteins which possess a MANEC domains with an e-value below 1E-5

NSP5 is characterized by a “motif at N terminus with eight cysteines” (MANEC) domain. However, the domain is present in the middle of the protein rather than at the N terminus (Fig. 5a). The MANEC domain is traditionally assumed to play a role in the formation of protein complexes, based on its structure [36]. Indeed, this domain is found in numerous membrane and extracellular proteins of multicellular animals. Although MANEC proteins are widespread among animals, BLAST searches indicated that only cnidarian sequences shared the same domain organization. All other non-cnidarian sequences with an e-value below 1e-05 were much longer and included additional protein domains. Interestingly, the presence of transmembrane domains was predicted in *Kudoa* (at the C-terminal end), *Enteromyxum* (at the N-terminal end) and *Polypodium* (at both N and C-terminal end), but not in other NSP5 sequences. Similarly, about half of the cnidarian outgroup sequences also possess a transmembrane domain at their C-terminal end. Because several sequences are truncated, this proportion is likely to be higher among complete sequences, which supports the view that NSP5 is a membrane protein. None of the NSP5 sequences were predicted to possess a signal peptide.

#### Phylogeny

Many cnidarian MANEC-containing genes were recovered by BLAST searches, but only a subset clustered with the nematocyst sequence of *Hydra* in a cnidarian-specific clade with high support (BP = 95 / PP = 1.0), which we are calling NSP5 (Fig. 5b). Relationships among Anthozoa, Medusozoa, and Myxozoa, were poorly resolved (BP < 70 PP < 0.5), and did not agree with the standard view of cnidarian relationships. A long-branch attraction artifact is probably responsible for the disagreement since the fast-evolving Myxozoa are placed at the base of the NSP5 clade and rooting the NSP5 gene with anthozoan would recover the traditional relationships.

### NSP6

#### Structure

**Fig. 6:**
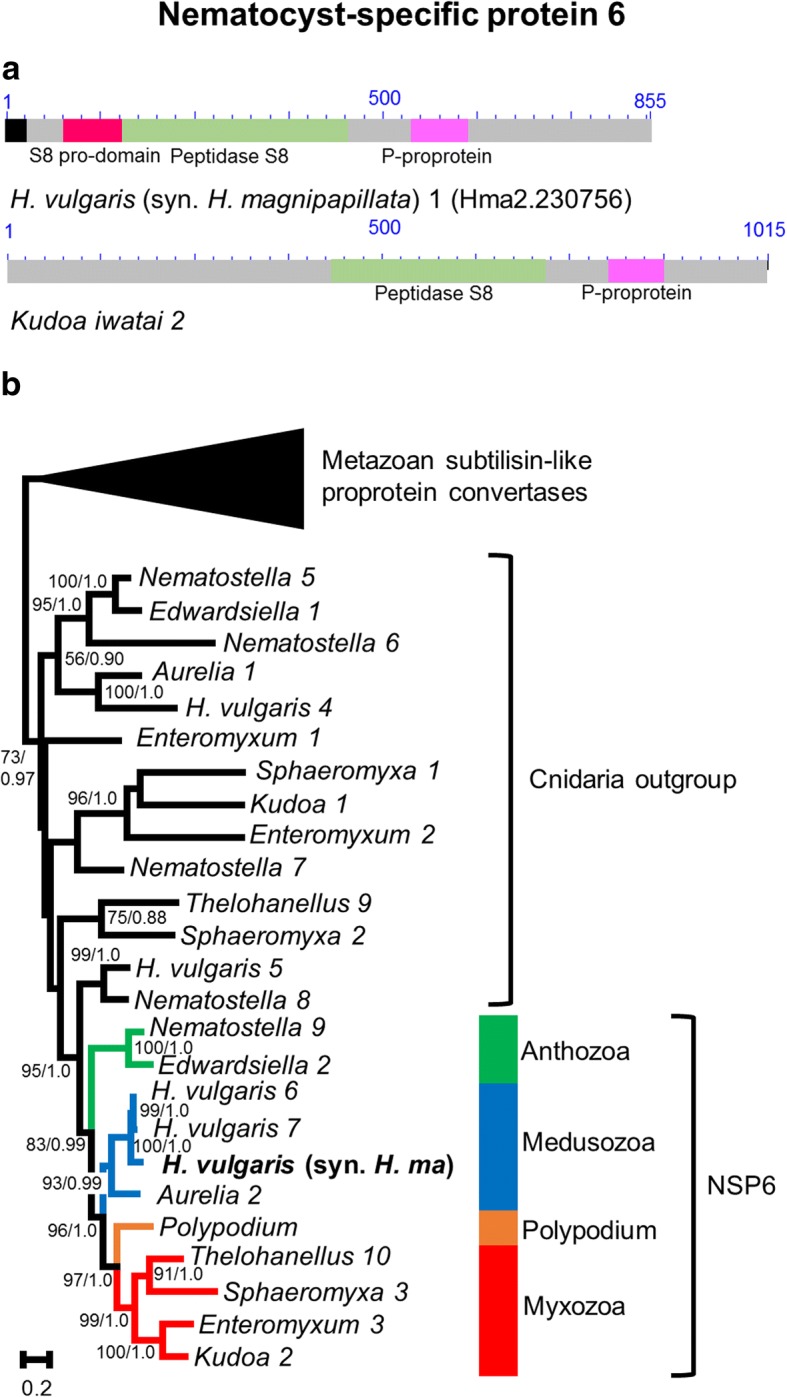
Nematocyst-specific protein 6 domain architecture and ML phylogenetic tree. **a**. Schematic drawing of the domain architecture of NSP6 for *Hydra magnipapillata* and *Kudoa iwatai*. For each domain the CDD definition is given. Signal peptides found are colored in black. **b**. ML phylogenetic tree reconstructed using the NSP6 dataset. ML bootstrap (BP_ML_)/Bayesian posterior probabilities supports are given for nodes with BP_ML_ above 50%. Red, orange, blue and green represent Myxozoa, *Polypodium*, Medusozoa, and Anthozoa respectively. To simplify the figure, outgroup sequences, which consist of metazoan subtilisin-like proprotein convertases, are represented by a triangle. The complete tree is available as Additional file 1. The original *H. vulgaris* (syn. *H. magnipapillata*) protein appears in bold. The tree was rooted with distant animal and cnidarian sequence with an E-value below 1E-05

The *Hydra vulgaris* (syn. *H. magnipapillata*) NSP6 protein is composed of three domains: a peptidase S8 pro-domain, a peptidase S8 domain, and a P-proprotein domain (Fig. 6a). These three domains are frequently associated in members of the peptidase S8 or subtilisin family of proteases. Subtilisins form a large family of serine proteases that are present in all domains of life [37]. This suggests that NSP6 originated from gene duplication and was co-opted to the nematocyst. A signal peptide was detected in *Hydra* and *Polypodium* but not in other proteins, which probably have a truncated N-terminal end.

#### Phylogeny

The NSP6 clade is nested among subtilisin-like proprotein convertases members, with high support (BP = 83; PP = 1.0) (Fig. 6b, Additional file 1). It includes members of all major clades of Cnidaria (i.e., Anthozoa, Medusozoa, Myxozoa and *Polypodium*). Phylogenetic relationships agree with the current view of cnidarian relationships and with the presence of additional recent duplications in *H. vulgaris* (in which 3 copies of the gene have been reported).

### NSP7

#### Structure

**Fig. 7:**
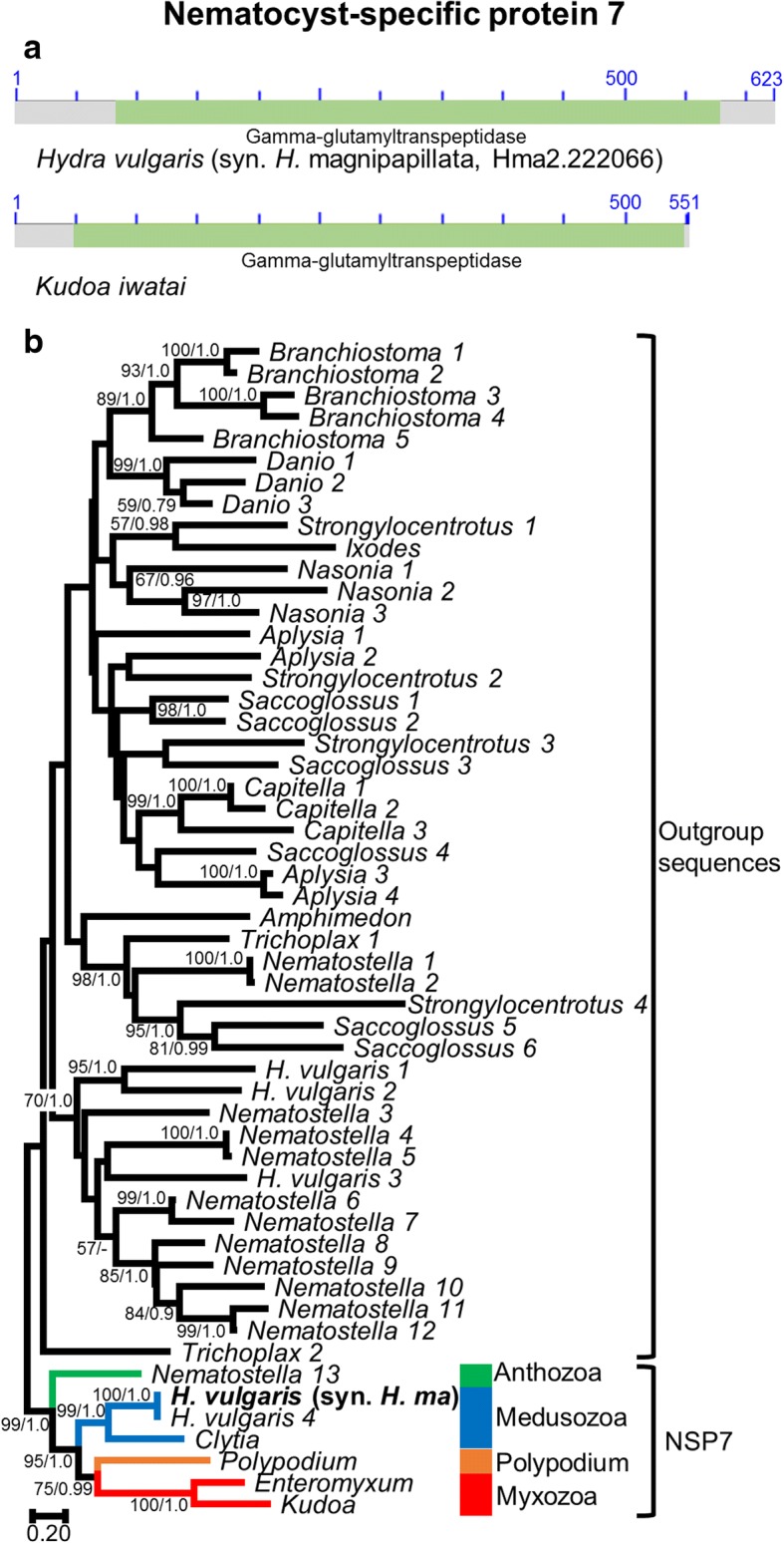
Nematocyst-specific protein 7 domain architecture and ML phylogenetic tree. **a**. Schematic drawing of the domain architecture of NSP7 for *Hydra magnipapillata* and *Kudoa iwatai*. For each domain the CDD definition is given. **b**. ML phylogenetic tree reconstructed using the NSP7 dataset. ML bootstrap (BP_ML_)/Bayesian posterior probabilities supports are given for nodes with BP_ML_ above 50%. Red, orange, blue and green represent Myxozoa, *Polypodium*, Medusozoa, and Anthozoa respectively. The original *H. vulgaris* (syn. *H. magnipapillata*) protein appears in bold. The tree was rooted with distant animal and cnidarian sequence with an E-value below 1E-05

NSP7 is composed of a single gamma-glutamyltranspeptidase domain (Fig. 7a). Gamma-glutamyltranspeptidase catalyzes the transfer of a gamma-glutamyl group from glutathione to an acceptor that can be an amino acid or a peptide [38]. No signal peptides were detected, except for *Polypodium,* which could represent a false positive. A transmembrane region was identified at the beginning of the gene in all species except for Myxozoa.

#### Phylogeny

While gamma-glutamyl transpeptidase is a large family whose evolution is characterized by numerous lineage-specific duplications, the NSP7 clade is composed of a single copy protein in all cnidarian species considered. Homologs of NSP7 were found in representatives of all major clades of Cnidaria (i.e., Anthozoa, Medusozoa, Myxozoa and *Polypodium*), and the NSP7 tree supports the current view of cnidarian relationships (Fig. 7b) [9, 26].

## Discussion

### Characterization of nematocyst-specific genes

Previous comparative studies of nematocyst protein content have focused on soluble proteins which encompass the venom proteins (e.g., [13, 39–41]). Specifically, Rachamim et al. [13] showed that out of 291 *H. magnipapillata,* 737 *A. aurita*, and 374 *Anemonia viridis* soluble nematocyst proteins present, only 6 were shared between these three species. This indicates weak conservation among proteins involved in the injectable content of the nematocyst. By contrast, because all nematocysts share a similar structure [42] we expected that several shell protein might be conserved among cnidarians. Our hypothesis was supported by our previous finding regarding the presence of minicollagens and nematogalectins in Myxozoa [11].

Thus far, the entire nematocyst proteome, which also include collagenous proteins which form the nematocyst shell, has only been characterized for *H. vulgaris* [14] and recently for the myxozoan *Ceratonova shasta* [17]. These two studies revealed a difference in protein numbers, with 410 unique proteins in *Hydra* and only 112 in *Ceratonova.* However, it is worth noting that the soluble content of the *Hydra* nematocyst is known to contain ~ 300 proteins [13] while the shell proteome represents only ~ 100 proteins, including ~ 20 minicollagen genes [14]. In agreement, the polar capsule proteome of *C. shasta* is expected to represent mainly shell proteins since *C. shasta* polar capsules are incapable of injection and do not contain venom proteins [17].These numbers suggest that the nematocysts shell is composed of about a hundred proteins, among which only minicollagens and nematogalectins have been recognized as core proteins shared by all cnidarian lineages.

This study has identified and characterized seven genes that are present in representative cnidarian taxa, including myxozoans. However, the methods used did not allow us to determine the location of these proteins in the cell. Interestingly, five of the seven genes (NSP1–3, NSP6–7) were identified in the polar-capsule proteome of *C. shasta,* a species that is closely related to *Kudoa* and *Enteromyxum* [17] (Table 1). Because the *C. shasta* sequences have not been submitted to public databases and are only available in the supplementary material of Piriatinskiy et al. (2017), they were not included in our phylogenetic analyses. However, the fact that most of the NSPs characterized from *Hydra* nematocysts are also present in the polar-capsule of *C. shasta*, strongly suggests that the nematocyst function of these NSP genes is conserved in all Cnidaria.

It is interesting to note that the majority of the nematocyst-specific cnidarian-restricted genes characterized here are structural and/or shell proteins. This illustrates that the conserved nature of nematocysts across Cnidaria is primarily in the structure of the capsule and tubule and not in the venom and/or enzymatic properties included in the injectable content of the nematocyst, when present. Indeed, not all nematocysts possess an injectable content, as some of them, such as desmonemes, are involved in prey attachment [43]. In NSP 2, 3, 4, and 6 a signal peptide was found in untruncated proteins (Table 1), indicating that these proteins are intended for the ER/Golgi secretory pathway [44]. This agrees with observations that the nematocyst is formed by the fusion of post-golgi vesicles [31].

### Evolutionary origins of nematocyst-specific genes

Although nematocyst proteins have been isolated and characterized in a few Cnidaria [13, 14, 16, 17], hitherto only minicollagens and nematogalectins have been characterized in a phylogenetic context [6, 11, 18]. Interestingly, out of the seven cnidarian-specific genes characterized here, five had never previously been identified as cnidarian-restricted.

Four of the seven NSPs appear to be “orphan” proteins, meaning that they do not easily demonstrate any clear similarities with proteins in other animals. However, six of the seven genes possess conserved functional domains that are also found in other metazoans, suggesting that the most likely origin of these genes is that of domain duplication and exon-shuffling. Two of the six (NSP6–7) possess paralogous copies in taxa outside of Cnidaria, indicating that they originated from gene duplication and neofunctionalization. Only NSP4, which is the shortest gene, possesses no similarity to any known domain or gene. It is, consequently, the only gene that may have had a *de-novo* origin in the cnidarian ancestor.

Our results thus suggest that the origin of nematocyst proteins in the ancestor of cnidarians was primarily through genome re-arrangements of existing genes/domains, as opposed to the evolution of *de-novo* genes.

### Limits to the identification of nematocyst-specific, cnidarian-restricted genes

Many of the genes that we characterized here, as well as some that have been previously reported, have undergone gene or exon duplication. Appropriate comparisons require the establishment of orthology through phylogenetic analysis.

It is worth noting that we took a very conservative and stringent approach and thus there are probably many other nematocyst-specific proteins in Cnidaria. In addition, myxozoans are highly derived and have a fast rate of DNA evolution [9]. Since we focused on nematocyst genes that are present in Myxozoa, our criteria probably precluded the identification of additional existing NSPs, due to extreme sequence divergence. Additionally, our BLAST searches began by using only *Hydra* sequences as queries, restricting our analyses to those only found in *Hydra* and myxozoans. As a case in point Piriatinskiy et al. [17] noted that proteins with a Wall Stress-responsive Component (WSC) domain (i.e., a putative carbohydrate binding domain) are present among the nematocyst proteome of myxozoan. Although our reciprocal BLAST searches identified such proteins, support values in the phylogenetic analyses were low and thus are not presented here.

Similarly, NSP diversity might be more important within specific cnidarian lineages. Hydrozoans, in particular, are known to possess a wide variety of nematocyst types in comparison to anthozoans [31, 43, 45, 46]. Indeed, it has been assumed that the large diversity of minicollagen genes observed in hydrozoans matches the diverse nematocyst repertoire of this group [31], i.e., different minicollagens are expressed in different nematocysts. Interestingly, the larger diversity of minicollagen and nematogalectin transcripts observed in *Hydra* originated from tandem duplications of exons that are alternatively spliced while the peptide signal is conserved [15], a pattern that is shared with NSP2. Because transcripts that share part of their sequence might be incorrectly assembled from short-read libraries, it is possible that such duplications might be overlooked, leading to an underestimation of the number of NSPs.

Finally, although there is some overlap in our identification of cnidarian-restricted nematocyst-specific genes, our results differ from previous studies [2, 17, 24, 25]. This is largely due to the fact that previous studies used sequence similarities in BLAST searches and did not apply a phylogenetic criterion. Our analyses demonstrate that reciprocal BLAST searches are inadequate for the identification of lineage-restricted genes. Phylogenetic reconstructions are necessary to determine whether putative lineage-restricted genes form their own clade, exclusive of genes from other lineages. For example, several of the genes characterized as NSP were not considered as exclusive to Cnidaria by Balasubramanian et al. [14] (Table 1) since other animal lineages possess paralogous protein domains with sequence similarity. Our phylogenetic analyses allowed us to determine ancient gene duplications and to characterize well-supported cnidarian-only clades of genes (see Methods for details).

### Nematocyst-specific genes provide insight into myxozoan evolution

Myxozoa polar capsules have been found to possess physical characteristics that differ from other nematocyst. Specifically, myxozoan polar tubules possess the ability to contract which is absent in other nematocysts [47]. This contraction mechanism has been proposed to be an adaptation to parasitism, since it facilitated the contact with the host by pulling the spore towards the host [47]. In addition, the ability to inject seems to have been either lost [17] or modified [47].

While polar capsules evolved to specialize in spore attachment, we show that they still retain several cnidarian specific proteins. Specifically, in addition to previously published minicollagens and nematogalectins [26], there are at least seven other nematocyst-specific genes that are shared by myxozoans and other cnidarians. This further confirms the position of Myxozoa as part of Cnidaria and the homology between the myxozoan polar capsule and the cnidarian nematocyst. Given that myxozoans lack nearly all evidence of a cnidarian origin, the nematocysts, and the genes that encode them, are a critical source of information for the investigation of myxozoan origins and evolution.

## Conclusions

This study has identified and characterized seven cnidarian-restricted genes present in several cnidarian taxa, including myxozoans. Our BLAST results, in conjunction with phylogenetic analyses, revealed that four of these genes do not possess any known orthologs in taxa outside of Cnidaria. Four of the seven genes have never previously been identified as cnidarian-restricted and none have previously been characterized in a phylogenetic context to determine homology. These findings significantly increase our understanding of the conserved molecular composition of nematocysts across Cnidaria.

## Methods

### Reciprocal BLAST searches in *Kudoa* transcriptome and genome

We downloaded the entire proteome of the *H. vulgaris* (syn. *H. magnipapillata*) nematocyst (329 proteins characterized by tandem mass spectrometry (MS\MS)) [14]. These proteins were used as a query to conduct first tblastn searches against the transcriptome and genome of *K. iwatai* with a *p*-value cutoff of 1e-05 [48]. *K. iwatai* was chosen as the myxozoan representative because it has a relatively complete genome and transcriptome [6]. We then ran a reciprocal blastx search using only the first hits as query against the entire proteome of *H. vulgaris* (syn. *H. magnipapillata*) (see Additional file 2 for details concerning the source of the proteome sequences) with the same cutoff, and kept only proteins that returned the *H. vulgaris* protein that had been used as query in the first search. Twenty-six sequences were selected at this stage and translated into proteins.

### Preliminary phylogenetic analyses

For each of the 26 myxozoan protein hits, a sequence alignment was built with exemplars of metazoan diversity. The metazoan species chosen were *Danio rerio*, *Branchiostoma floridae*, *Saccoglossus kowalevskii*, *Strongylocentrotus purpuratus*, *Nasonia vitripennis*, *Ixodes scapularis*, *Capitella teleta*, *Aplysia californica*, *Trichoplax adhaerens*, and *Amphimedon queenslandica.* In addition we also searched all cnidarian proteins present in the protein database of NCBI (taxid: 6073) (last accessed 29 November, 2015). We also searched the proteome of *H. vulgaris* and the genome and transcriptome of *K. iwatai* for the presence of duplicates that would not have been identified in the reciprocal BLAST searches.

For the initial round of phylogenetic analyses we compiled datasets from blastp searches on NCBI (last performed on the NCBI database in November 2015) with both *Hydra* and myxozoan proteins as query against the NCBI database for the species indicated above, and downloaded all proteins with an e-value below 1e-05. Some of the proteins downloaded had a very different domain organization than the *Hydra* and *Kudoa* sequences, which affected the reliability of the sequence alignments. In order to eliminate the most distant protein sequences with different domain organization we excluded all hits that were either at least twice the length of the *Hydra* protein query, which suggested the presence of additional domains. Similarly, we excluded all proteins shorter than 100 aa. Of note, all *Hydra* proteins considered were longer than 200 aa. Identical sequences from the same species were also excluded from the analyses.

The resulting datasets from the blastp searches were aligned using MAFFT version 7 [10] under the L-ins-I algorithm [49]. We did not exclude any positions at this stage to ensure a better identification of the main clades. Preliminary phylogenetic trees were created using RaxML 8.0.26 under the options ML + rapid bootstrap, 100 bootstrap, PROTGAMMA, LG + F [50].

Myxozoa proteins with a cnidarian-specific origin were identified for further phylogenetic analyses as those proteins that included only cnidarian sequences as identified from the blastp searches, or those that were duplicated in cnidarians. Duplicated cnidarian genes had to form at least two clades, one of which had to be a highly supported cnidarian specific clade that includes the reference *Hydra* nematocyst protein sequence and the *Kudoa* sequence. Seven proteins were found to follow these criteria.

### Final phylogenetic analyses

The cnidarian-specific origins of the seven proteins was then confirmed using a larger taxonomic sampling and more thorough phylogenetic analyses. Following the preliminary phylogenetic analyses (see above), we expanded our dataset to include publicly available transcriptome data from the myxozoans *K. iwatai*, S*. zaharoni*, *E. leei* and *T. kitauei* [51], and other cnidarians *Clytia hemisphaerica*, *Acropora millepora*, *Aurelia aurita*, *Pocillopora damicornis*, and *Edwardsiella lineata* (Additional file 2). The criteria described in the above paragraph were used to select sequences based on sequence length and an E-value cut-off of 1e-05. Additionally, in the final tree analyses we also excluded proteins that presented a different domain organization (i.e., they included at least one different domain or presented domain duplications that were not compatible with the *K. iwatai* or *H. vulgaris* organization). Truncated sequences that did not depart from the *Hydra* domain organization were however included, even if they missed some of the domains. Searches were also performed against the ESTs of *Buddenbrockia* [26] and *Tetracapsuloides* [24], but no BLAST hits were obtained. The absence of polar capsule genes is most probably an artefact of incomplete transcriptome data of these two species, as evident when compared to other Myxozoan sequence data (see, for example, dataset S4 in [9]). Similarly, we failed to identify any of the NSP genes within the filtered transcriptome of *Myxobolus pendula* as available in the supplementary material of Foox et al. [19]. This may come from the stringent filtration pipeline used by the authors to characterize myxozoan transcripts from contaminants [19].

BLAST searches were performed using the *H. vulgaris* (syn. *H. magnipapillata*) and *K. iwatai* proteins identified as indicated previously as query against the DNA assemblies. It should be noted that for some species sequences were assembled manually from several EST sequences. The sequence accession numbers are provided in Additional file 3. Intron-exon predictions were then performed for Myxozoa species with the Augustus web server [52]. We evaluated manually the different eukaryote model organisms, by aligning the predicted sequences to the *Kudoa* (transcriptome sequence) and the *Hydra* sequence. Our results indicated that the bee (*Apis mellifera*) model gave the best prediction while other models usually skipped exons or gave no result at all. Manual adjustment of the intron-exon boundaries were then performed by comparing the intron locations in *Kudoa* DNA and RNA and by comparing the proteins and locating missing spaces. We performed a domain search on all sequences using the NCBI’s CD-Search interface using default settings [53]. Putative signal peptides sequences were identified with SignalP 4.1 using the sensitive option [44]. Transmembrane domains were predicted with TMHMM Server v. 2.0 [54]. For the final phylogenetic analyses shown in Figs. 1, 2, 3, 4, 5, 6, 7, our dataset comprised the sequences that were identified in the BLAST searches and had no more than the described domains present.

Alignments were performed using MAFFT version 7 under the L-ins-i setting for proteins with a single protein domain, and under the E-ins-i setting for genes with more than one protein domain [49]. Positions with more that 50% of missing data were excluded from the alignment, and phylogenetic analyses were performed under the maximum likelihood (ML) and the Bayesian criterions. The alignments are provided in the supplementary material (Additional files 4, 5, 6, 7, 8, 9, 10). For each protein alignment, the program Prottest 3.2 [55] was run under the default settings [55]. The AIC was used to identify the best ML model. The model chosen was then used to reconstruct the tree using RaxML version 8.1.21 [50]. ML trees were computed using 50 starting trees, and bootstrap supports were computed using 1000 “thorough replicates” (option -f i). Bayesian analyses were conducted with the program MrBayes v3.2.6 [56] under the “mixed protein model” + Gamma. Two runs, with four chains each, were conducted under default temperature parameters and default prior distributions. Each chain was run for 15,000,000 generations and sampled every 100 generations. The burninfrac parameter was set to 0.25. Convergence was achieved before the end of the burnin for all markers.

## Additional files

Additional file 1: Complete ML phylogenetic tree reconstructed using the NSP6 dataset. ML bootstrap (BP_ML_)/Bayesian posterior probabilities supports are given for nodes with BP_ML_ above 50%. Red, orange, blue and green represent Myxozoa, *Polypodium*, Medusozoa, and Anthozoa respectively. The original *H. vulgaris* (syn. *H. magnipapillata*) protein appears in bold. The tree was rooted with distant animal and cnidarian sequence with an E-value below 1E-05. (PDF 28 kb)

Additional file 2: List of databases used in BLAST searches. For each species the data type used and the URL/NCBI database are indicated. (XLSX 11 kb)

Additional file 3: GenBank accession numbers of the sequences used in the phylogenetic analyses. The table indicates the species name and accession number for each sequence from Figs. 1, 2, 3, 4, 5, 6, 7. (XLSX 20 kb)

Additional file 4: NSP1 protein alignment. Protein sequence alignment, in Nexus format. (NEX 13 kb)

Additional file 5: NSP2 protein alignment. Protein sequence alignment, in Nexus format. (NEX 15 kb)

Additional file 6: NSP3 protein alignment. Protein sequence alignment, in Nexus format. (NEX 5 kb)

Additional file 7: NSP4 protein alignment. Protein sequence alignment, in Nexus format. (NEX 7 kb)

Additional file 8: NSP5 protein alignment. Protein sequence alignment, in Nexus format. (NEX 169 kb)

Additional file 9: NSP6 protein alignment. Protein sequence alignment, in Nexus format. (NEX 239 kb)

Additional file 10: NSP7 protein alignment. Protein sequence alignment, in Nexus format. (NEX 47 kb)
