# Supplementary figures and images for: A genome wide survey reveals multiple nematocyst-specific genes in Myxozoa
Source: BMC Evol Biol. 2018 Sep 12;18:138. doi: 10.1186/s12862-018-1253-7 (PMC6134521; doi:10.1186/s12862-018-1253-7)

# Nematocyst-specific protein 6

Metazoan subtilisin-like proprotein convertases

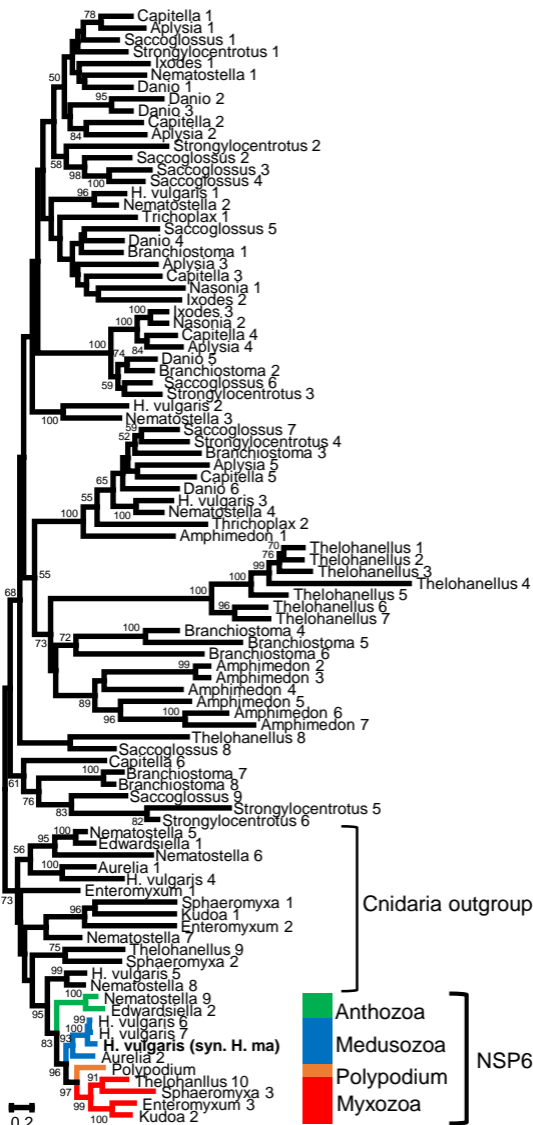

Supplement: Supplementary file 1 — Complete ML phylogenetic tree reconstructed using the NSP6 dataset. ML bootstrap (BPML)/Bayesian posterior probabilities supports are given for nodes with BPML above 50%. Red, orange, blue and green represent Myxozoa, Polypodium, Medusozoa, and Anthozoa respectively. The original H. vulgaris (syn. H. magnipapillata) protein appears in bold. The tree was rooted with distant animal and cnidarian sequence with an E-value below 1E-05. (PDF 28 kb) [file 12862_2018_1253_MOESM1_ESM.pdf]
